# The Ins and Outs of Autophagy and Metabolism in Hematopoietic and Leukemic Stem Cells: Food for Thought

**DOI:** 10.3389/fcell.2018.00120

**Published:** 2018-09-26

**Authors:** Angela Ianniciello, Kevin M. Rattigan, G. Vignir Helgason

**Affiliations:** Wolfson Wohl Cancer Research Centre, Institute of Cancer Sciences, University of Glasgow, Glasgow, United Kingdom

**Keywords:** HSCs, LSCs, autophagy, mitophagy, metabolism, quiescence

## Abstract

Discovered over fifty years ago, autophagy is a double-edged blade. On one hand, it regulates cellular energy sources by “cannibalization” of its own cellular components, feeding on proteins and other unused cytoplasmic factors. On the other, it is a recycling process that removes dangerous waste from the cytoplasm keeping the cell clean and healthy. Failure of the autophagic machinery is translated in dysfunction of the immune response, in aging, and in the progression of pathologies such as Parkinson disease, diabetes, and cancer. Further investigation identified autophagy with a protective role in specific types of cancer, whereas in other cases it can promote tumorigenesis. Evidence shows that treatment with chemotherapeutics can upregulate autophagy in order to maintain a stable intracellular environment promoting drug resistance and cell survival. Leukemia, a blood derived cancer, represents one of the malignancies in which autophagy is responsible for drug treatment failure. Inhibition of autophagy is becoming a strategic target for leukemic stem cell (LSC) eradication. Interestingly, the latest findings demonstrate that LSCs show higher levels of mitochondrial metabolism compared to normal stem cells. With this review, we aim to explore the links between autophagy and metabolism in the hematopoietic system, with special focus on primitive LSCs.

## Maintenance in the Niche

### Hypoxia, a Key Role in the Regulation of HSCs Quiescence

Decades of research has allowed scientists to characterize and describe the unique role of hematopoietic stem cells (HSCs) in the lifelong homeostasis of mature blood cells. The mammalian hematopoietic system is maintained by self-renewal of quiescent long-term (LT)-HSCs, which subsequently can differentiate into short-term (ST)-HSCs or multipotent progenitors (MPPs). The latter two will commit to either myeloid or lymphoid lineages exclusively. Because of their vital and long-term function, HSCs are provided with unique survival mechanisms. In this context, their localization in a complex endosteal niche, that is characterized by low levels of oxygen, is central (Szade et al., 2017). Changes in the bone marrow niche, such as increased production of growth factors and cytokines, as well as transplantation procedures and injuries, can stimulate HSCs to proliferate and differentiate. Once recovery is restored, HSCs return to a dormant state. Despite the fact that hypoxia tolerance in HSCs is poorly understood, it has been proposed to be responsible for the quiescence and slow cell cycling of HSCs (Cipolleschi et al., 1993; Parmar et al., 2007). Takubo et al. (2010) elucidated this mechanism by demonstrating that hypoxia-inducible factor-1α (HIF-1α), a transcriptional factor that plays a central role in cellular response to oxygen levels, is stabilized by hypoxia in LT-HSCs. In HIF-1α deficient mice, loss of LT-HSCs numbers is directly proportional to loss of quiescence. This now raises the question: in what ways hypoxia can assume a protective role and assure LT-HSCs maintenance? HIF-1α and hypoxia have in fact, been linked with the distinct metabolic phenotype of HSCs.

### HIF-1α and the Regulation of HSCs Metabolism

**FIGURE 1:**
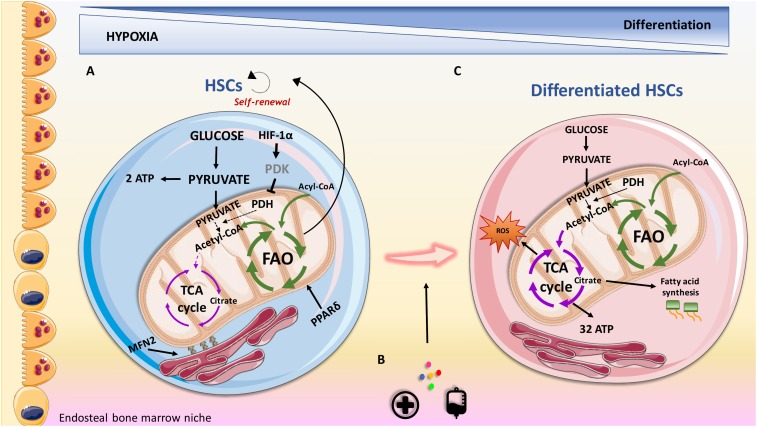
Overview of metabolic features contributing to HSCs maintenance and differentiation in the endosteal niche. **(A)** HSCs are characterized by the expression of the surface marker CD34 and utilize anaerobic glycolysis and fatty acid oxidation (FAO) as the sources of energy. Nutrient sensor peroxisome proliferator-activated receptor delta (PPARδ) promotes FAO, contributing to their self-renewal capacity. HIF-1α represents a fundamental feature in regulating oxidative metabolism via regulation of pyruvate dehydrogenase kinase (PDK). **(B)** Transplantation procedures, cytokines and injuries can promote HSCs to proliferate and differentiate. **(C)** Differentiated HSCs lose their stemness surface marker CD34 and adapt their energy source to address energy demand for proliferation. CD34^-^ HSCs, rely on increased oxidative metabolism that provides a higher production of ATP compared to anaerobic glycolysis. Oxidative metabolism can result in increased production of reactive oxygen species (ROS), which can contribute to differentiation. PDH, pyruvate dehydrogenase; MFN2, mitofusin 2.

Metabolomic approaches indicate that LT-HSCs, when compared to MPPs and more differentiated cells, specifically upregulate glycolysis and represses influx of glycolytic metabolites into mitochondria, via regulation of pyruvate dehydrogenase kinase (PDK) activity by HIF-1α (Takubo et al., 2013; **Figure 1**). Furthermore, glycolytic adenosine triphosphate (ATP) production, commissioned by the HIF-1α/PDK regulatory system, is necessary to maintain HSCs during cell cycle quiescence. A recent discovery demonstrates that mitochondrial protein mitofusin 2 (MFN2), which has roles in mitochondrial fusion and in tying mitochondria to the endoplasmic reticulum, is essential for the maintenance of HSCs with wide lymphoid potential (Luchsinger et al., 2016). A different study identified that the mitochondrial unfolded protein response (UPRmt) is activated upon transition from quiescence to proliferation in HSCs (Mohrin et al., 2018). Remodeling the activity of sirtuin 7 (SIRT7), a component of the UPRmt, is translated into reduction of quiescence, higher mitochondrial unfolded protein stress, and insufficient self-renewal ability of HSCs. Furthermore, SIRT7 expression is lower in more mature HSCs whose regenerative capacity is improved following upregulation of SIRT7 (Mohrin et al., 2015). Kim et al. (1998), using a cationic fluorescent dye that selectively accumulates in the mitochondria of eukaryotic cells, demonstrated that HSCs have relatively less mitochondria when compared to proliferating progenitors. A subsequent study showed that differentiation of primary human HSCs (quantified by CD34 loss) is connected with increased mitochondrial content (Piccoli et al., 2005). In agreement with this, Simsek et al. (2010) showed that LT-HSCs are characterized by low mitochondrial potential and utilize cytoplasmic glycolysis for ATP production. Contrarily, cells that need to cycle and expand do not rely on anaerobic glycolysis. This may be because pyruvate produced during glycolysis will generate only 2 ATPs per molecule of glucose following anaerobic respiration (with lactate being the by-product), while it will produce 32 ATPs per molecule of glucose upon entering in the mitochondria to be used for oxidative phosphorylation (OXPHOS). However, de Almeida et al. (2017) using dye-independent methods, such as mitochondria DNA quantification and enumeration of mitochondria nucleoids, have recently suggested that while HSCs have high mitochondrial content they have compromised respiratory and turnover capacity, concluding that mitochondria perform an essential and yet unknown function in HSCs, which may not be directly linked with ATP production.

### Stem Cell Proliferation and Maintenance, a Key Role for Fatty Acid Oxidation

LT-HSCs glycolytic phenotype can be seen as a protective mechanism to reduce reactive oxygen species (ROS) generation, which would cause oxidative stress and induce differentiation (Folmes et al., 2012). Based on this, a recent study demonstrates that while mitochondrial complex III subunit Rieske iron sulphur protein (RISP) in fetal HSCs is not essential for mitochondrial membrane potential maintenance, it is crucial for stem cell genes and multilineage potential retainment (Ansó et al., 2017). Based on the critical role of mitochondria in driving cell differentiation, RISP null fetal HSCs were unable to generate an adequate number of MPPs indicating compromised HSCs differentiation. In addition, products of the tricarboxylic acid (TCA) cycle, such as citrate, could be exported to the cytosol to contribute to lipid metabolism that is required for cell growth, proliferation and differentiation (Lum et al., 2007).

HSCs fate is dictated by their decision to undergo symmetric or asymmetric cell division when HSCs leave quiescence. Asymmetric division generates two daughter cells of which one will show same features of the initiator cell, such as self-renewal and quiescence, and the other will differentiate and enter the circulatory system. Otherwise, symmetric division will generate two daughter cells that will only be able to undergo cell cycling and differentiation. Fatty acid oxidation (FAO), which occurs in the mitochondria, also plays an important role in HSCs maintenance. FAO metabolism prevents HSCs exhaustion when HSCs proliferation and division are required, resulting in asymmetric division and thus assuring self-renewal (Ito et al., 2012). A role for peroxisome proliferator-activated receptor delta (PPARδ), which is a member of a nuclear receptor superfamily of transcription factors that controls nutrient sensing and FAO, has been reported in HSCs. PPARδ deletion or pharmacological inhibition of FAO stimulates the symmetric commitment of HSCs leading to stem cell depletion, while PPARδ activation, via use of an agonist, increased asymmetric cell division. In agreement with this, a subsequent study showed that weakening the mitochondrial phosphatase protein tyrosine phosphatase 1 (PTPMT1), drives the conversion from glycolysis and FAO to mitochondrial aerobic metabolism, resulting in unsuccessful hematopoiesis (Yu et al., 2013). This is linked with accumulation of HSCs unable to differentiate due to increased entry of quiescent stem cells into the cell cycle and a following pause at the G1 phase.

## Hscs Maintenance: Glycolysis Versus Oxphos

As introduced above, HSCs maintenance is affected by a balance between HSCs metabolic status and ROS levels. In fact, dormant HSCs seem to rely on glycolysis to avoid a decline in HSCs maintenance and HSCs with defective glycolysis will switch to a mitochondrial metabolic profile with increased production of ROS (Wang et al., 2014). ROS have been largely found to contribute to bone marrow failure as one of the main sources for DNA damage and genome instability (Richardson et al., 2015), thus ROS can play a role as sensor dictating HSCs fate. Mitochondria, which are the main source of energy and indirectly of ROS, are considered as minor player in the maintenance of HSCs maintenance. However, HSCs highly rely on mitochondria when a metabolic switch is required (i.e., HSCs need to increase their proliferation rate). FOXO3, a transcriptional factor that shows multiple functions associated with longevity, is a regulator of HSCs metabolism. Loss of FOXO3 alters mitochondria function, inducing deleterious accumulation of ROS (Rimmele et al., 2015; Bigarella et al., 2017). Specifically, deletion of FOXO3 in HSCs compromise DNA repair pathway leading to DNA damage, which compromises HSCs function. Mutation in tuberous sclerosis complex 1 (TSC1), a negative regulator of mTOR and a key regulator for cellular metabolism, induces levels of ROS and loss of quiescence in HSCs (Chen et al., 2008). Additionally, mTOR activity contributes to erythroid differentiation favoring mitochondria activity and is also increased with aging (Luo et al., 2014; Liu et al., 2017). Moreover, mTOR is one of the main regulators of autophagy, a process that itself has a critical role in HSCs biology.

## Autophagy

Autophagy, from the Greek *auto*-self and -*phagy* eating, is an evolutionally conserved process first described in yeast in 1963 by Christian de Duve (de Reuck, 1963). It is a lysosomal catabolic process that has several functions. First of all, it has a role as a cell cleaner by reducing the chance of cell misfunction due to accumulation of damaged cellular components and organelles. It is also involved in microbe’s demolition and sustains metabolism during stressful situations, such as starvation, providing building blocks for energy production and cellular homeostasis. The assembly of the catabolic machinery of autophagy takes place in the cytoplasm, in double membrane vesicles known as autophagosomes. Numerous autophagy-related (*ATG*) genes are involved in their biogenesis and function that can be organized in three main stages (Mizushima et al., 2011). The very first step consists in the autophagy initiation and formation of the phagophore. Signals of cellular nutrient status are sensed by the unc-51 like autophagy activating kinase 1 (ULK1) initiation complex which then activates autophagy and recruits a second complex, known as VPS34 complex, resulting in the formation of a flat unique membrane known as phagophore (Ganley et al., 2009; Russell et al., 2013). Phagophores will then elongate and expand leading to autophagosomal maturation. The last step is represented by their fusion with lysosomes where proteases will be in charge of their content demolition (Kim et al., 2002; Yang and Klionsky, 2009). Although, keeping LT-HSCs in their hypoxic niche seems to satisfy maintenance of their “dormant” state, it may not be the only factor that contributes to their metabolism adaptation. Lately, autophagy has been shown to be essential in preserving the organization and the welfare of this small cell compartment (Warr et al., 2013b; Nguyen-McCarty and Klein, 2017). The maintenance of cell health and prevention of stem cell aging is also vital for hematopoiesis, and the role of autophagy in degrading damaged cellular components and organelles may be essential in this context. Furthermore, autophagy flux negatively correlates with cell decline in several cell subtypes including HSCs (Revuelta and Matheu, 2017).

### Autophagy, Key Player in HSCs Maintenance

The fact that HSCs have a slow turnover increases the chance to reduce or dilute damaged cellular components and autophagy might be indispensable for the necessary increase in catabolic rate. Several studies propose that autophagy can sustain glycolytic flux, protecting HSCs from metabolic stress and expansion stimulus in the bone marrow, thereby reducing the chance of HSCs exhaustion. FOXO3a, a transcriptional factor that shows multiple functions associated with longevity, regulates levels of autophagy in HSCs in case of metabolic stress (Warr et al., 2013a). Specifically, FOXO3a deficient mice showed a pronounced reduction in autophagy capacity in protecting HSCs. FIP200, a component of the ULK1 initiation complex, has also been associated with maintenance of HSCs (Liu et al., 2010). This study found that mice depleted with FIP200 resulted in increased HSC proliferation, in which mitochondrial mass was higher when compared with HSC with no depletion. ATG7, whose role is only associated with autophagy, regulates mitochondrial homeostasis of HSCs, as well as ROS production and cell differentiation (Mortensen et al., 2011). Deleting ATG7 in the hematopoietic compartment results in the loss of normal HSCs functions and severe myelo-proliferation, causing mice death. The authors also show that the hematopoietic stem and progenitor cell (HSPC) compartment exhibit accumulation of mitochondria and ROS, in addition to an increased proliferation rate and DNA impairment.

### Autophagy and HSCs Cell Cycle

**FIGURE 2:**
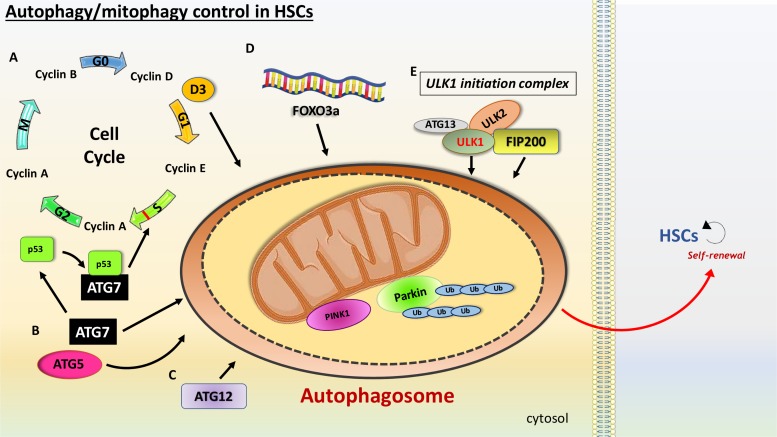
Schematic view of the main features involved in the autophagy promotion in HSCs. HSCs are characterized with high levels of autophagy. **(A)** Slow cell cycle is essential to ensure maintenance of HSCs. Cyclins are a family of proteins that control the progression of cells through the cell cycle. Cyclin D3, a member of the cyclin D family involved in the G1 phase of the cell cycle, is a nutrient stress sensor, promoting autophagy as a fuel source for cell cycle entry. ATG7 and ATG5 have been linked with mitophagy induction in HSCs. **(B)** ATG7 binds p53/TP53, an S phase checkpoint control that senses nutrient stress, modulating cell cycle exit. **(C)** ATG12 has also been linked with HSCs maintenance. **(D)** FOXO3a reduces HSCs exhaustions via regulation of autophagy. **(E)** Members of the ULK1 initiation complex, such as ULK1 and FIP200, have been linked with mitophagy in HSCs.

Orientating HSCs to quiescence and a slow cell cycle ensure preservation and health of long-lived stem cells. Fewer replication cycles with minimum telomere shortening ensure that HSCs age more slowly (Liu and Rando, 2011). Despite the fact that cyclin D family members, including cyclins D1–D3, are expressed at different levels in HSPCs, Cao et al. (2015) showed that only cyclin D3 responds to nutrient stress and identified autophagy as a driving force for cell cycle entry. Therefore, they suggest that the lower levels of autophagy activity observed in aged mice may be due to lower cyclin D3 levels, and thus a postponed HSCs entry into the cell cycle. This ultimately results in a defect in self-renewal (**Figure 2**). The most common way to test HSCs ability to undergo self-renewal is represented by serial transplantation of murine HSCs from an original donor to a recipient, whose own HSCs were completely ablated before transplantation. Recent studies indicate that autophagy is an essential process for self-renewal. Ho et al. (2017), demonstrated that mice with hematopoietic-specific deletion of the essential autophagy factor ATG12 had increased numbers of cells in the peripheral blood and spleen. The authors then performed serial transplantation of ATG12 deficient HSCs into recipient mice. The mice receiving the ATG12 deficient HSCs showed dramatically impaired engraftment and reduced number of regenerated HSCs. It has recently been reported that ATG7 can bind p53 and modulate TP53/p53 checkpoint in cell cycle exit in response to metabolic stress (Lee et al., 2012). Here authors showed that starved murine fibroblasts lacking ATG7 fail to undergo cell cycle arrest.

### Mitophagy Controls Oxidative Stress in HSCs

It is likely that to maintain latency in HSCs, it is fundamental to have low mitochondrial activity. A phenomenon known as mitophagy is the only known process for mitochondrial clearance, and this function has been demonstrated to control levels of oxidative stress. One of the key regulators of mitophagy is PTEN-induced putative kinase 1 (Pink1) that interacts with the outer mitochondrial membrane and with Parkin, a E3 ubiquitin ligase, guiding mitochondria to autophagosomal degradation (Michiorri et al., 2010; Baudot et al., 2015). In their study Jin et al. (2018) reported that ATAD3A is a major regulator of mitophagy. During mitophagy in the hematopoietic system, ATAD3A functions as a bridge between the translocase of the outer membrane complex and the translocase of the inner membrane complex to facilitate the import of Pink1 into mitochondria. Deletion of ATAD3A results in enhanced mitophagy with mitochondria depletion and blockage in differentiation, restoring HSCs pool. Another study showed that PPAR–FAO pathway mediates clearance of damaged mitochondria, an important process in the self-renewing population expansion of Tie2^+^ HSCs in mice (Ito et al., 2016). Tie2, a receptor tyrosine kinase, expression on HSCs is a marker of quiescence (Iwama et al., 1993; Arai et al., 2004). Additionally, ATG5 and ATG7 have been shown to regulate mitophagy and oxidative stress (Zhang et al., 2009). A robust increase in mitochondrial mass is detected in mice with conditional depletion of ATG5 or ATG7. This is translated in increased production of ROS and higher DNA damage in ATG7 deficient cells than that of their wild-type counterparts. Authors therefore highlight mitophagy as a critical mechanism for normal HSPC function. Furthermore, it has been reported that mice deficient in ULK1 have compromised mitochondrial clearance during the stages of erythrocyte maturation (Kundu et al., 2008). As previously mentioned, ROS have a major role in HSCs decline. Since mitochondria are the main source for ROS, mitophagy might also represent a crucial regulator for HSCs aging. Reducing oxidative stress via mitochondria degradation might therefore prevent HSCs exhaustion and immature aging, although further investigation to address this hypothesis is needed.

## Lscs and the Origin of Myeloid and Lymphoid Leukemia

The ground-breaking discovery of the HSCs niche was made by Schofield (1978). Since then, significant advances have been made in describing what orchestrates HSCs maintenance. Mutagenesis or epigenetic changes in HSCs, together with fluctuations in the bone marrow microenvironment, are important events to cause blood malignancies as leukemia. Based on functional and immunophenotypic investigation of various subtype of cells, the existence of cancer stem cells (CSCs) was firstly described in the hematopoietic system and it is proposed that leukemia is a stem cell disorder, initiated by as little as a single leukemic stem cell (LSC). These cells can originate either from rare transformed HSCs or from more abundant and more differentiated progenitor cells. The origin of LSCs can vary with the stage of the disease, whether the leukemia is chronic or acute, its immunophenotype, myeloid or lymphoid, and the nature of the transforming event. HSCs are equipped with intrinsic self-renewal activity that persists for the whole life of an individual. In this context, HSCs have much higher chance to accumulate mutations than less primitive cells, which are not as long lived. LSCs initiation and maintenance is based on enhanced self-renewal activity (Argiropoulos and Humphries, 2007; Wang et al., 2010). In the case of leukemic cells, they could potentially originate from more restricted progenitors by acquiring mutations that allow them to self-renew, or from HSCs that accumulate genetic and epigenetic changes that down-regulate cell death and increase their self-renewal capability. To fully understand the vast heterogeneity of the disease we will briefly introduce each type of leukemia.

### Origin and Aberrations in AML

**FIGURE 3:**
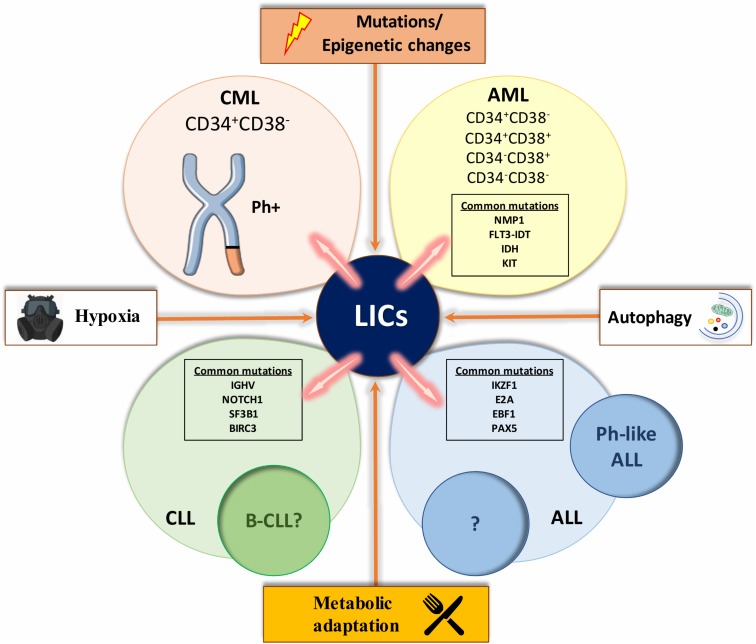
A compilation of factors involved in leukemic transformation. The figure represents a compilation of the various influences involved in the leukemic initiation process that characterizes each type of leukemia. Mutations and epigenetics changes, such as a distinct metabolic profile that drives leukemic stem cells (LSCs) expansion, autophagy which contributes to fuel LSCs energy demand and hypoxic environment, seem to be some of the main inducers of changes in HSCs and initiate leukemia. With the help of extended research in the field, we might be able to study and or perturb these influences for a better understanding of each type of leukemia and ultimately better-tailored therapeutics. List of abbreviations; CML, chronic myeloid leukemia; AML, acute myeloid leukemia; CLL, chronic lymphocytic leukemia; B-CLL, B cell CLL like phenotype; ALL, acute lymphoblastic leukemia; Ph-like ALL, Philadelphia chromosome-like ALL; Ph^+^, Philadelphia positive; *NPM1*, Nucleophosmin; *FLT3-ITD*, like tyrosine kinase 3-internal tandem duplication; *KIT*, proto-oncogene receptor tyrosine kinase; IKZF1, IKAROS family zinc finger 1; E2A, transcription factor 3; EBF1, early B-cell factor 1; PAX5, paired box 5; *IGHV*, non-mutated immunoglobulin heavy chain variable genes; *NOTCH1*, Notch homolog 1, translocation-associated; *SF3B1*, splicing factor 3B subunit 1; *BIRC3*, baculoviral IAP Repeat Containing 3. CD34 and CD38 are markers of hematopoietic stem and progenitor cells.

Acute myeloid leukemia (AML) is the most common leukemia in adults and the origin of AML has been thoroughly investigated. The *t*(8,21) and the *t*(15,17) are the most frequent chromosomal abnormalities associated with AML (Downing et al., 2000). Nucleophosmin (*NPM1*), CCAAT/enhancer-binding protein alpha (*CEBPA*), FMS like tyrosine kinase 3- internal tandem duplication (*FLT3-ITD*) and proto-oncogene receptor tyrosine kinase (*KIT*) are the most common mutations in AML patients, dictating the development of the leukemia and rearranging them into different prognostic groups (Welch et al., 2012; Yohe, 2015; **Figure 3**). Mutations such as isocitrate dehydrogenase (*IDH*) that occurs in early stages of the disease, lead to pre-leukemia development of AML (founder mutation) (Thomas and Majeti, 2017). Later in the disease progression, acquisition of driver mutations such as *FLT3-ITD* can cause the full-blown disease phenotype and further tertiary mutations can contribute to disease heterogeneity. In 1994 it was shown that leukemic cells possessing the CD34^+^CD38^-^ cell-surface markers were able to initiate leukemia in severe combined immunodeficiency (SCID) mice, while CD34^+^ or certain CD34^+^CD38^+^ expressing cells were unable to do so. Moreover, limiting dilution assays showed that leukemic-initiating cells (LICs) were a small fraction of the entire disease, representing roughly 1 in 250,000 leukemic cells (Lapidot et al., 1994). Bonnet and Dick, the pioneers of developing and refining transplantation techniques of human cells into recipient mice, demonstrated that only CD34^+^CD38^-^ fractions of cell types isolated from AML patients could engraft in recipient mice (Kamel-Reid et al., 1989; Lapidot et al., 1994). This observation has been further supported by the finding of Blair et al. (1997) indicating that LICs from human AML samples were also Thy-1^-^. However, Taussig et al. (2010) indicate that LICs from AML patients with mutated NPM1 reside in the CD34^-^ fraction.

### Origin and Aberrations in CML

Almost 100% of chronic myeloid leukemia (CML) patients are positive for the Philadelphia (Ph) chromosome, a shortened chromosome 22 that arises from a reciprocal translocation *t*(9q34,22q11) (Rowley, 1973; Raskind and Fialkow, 1987). The Ph chromosome is the hallmark of the disease, in which fusion of *BCR* and *ABL* genes encode for an constitutively active protein kinase (Daley et al., 1990; Sawyers, 1999). Since BCR-ABL fusion can occur in myeloid, B lymphoid, erythroid and sporadically T lymphoid cells in the majority of CML patients, the consensus is that the original translocation takes place in LT-HSCs (Fialkow et al., 1977). The presence of BCR-ABL in endothelial cells originating from CML patient, raises the question: does the aberration take place even in more primitive cells than LT-HSC (Gunsilius et al., 2000)? An elegant experiment conducted by Fialkow et al. (1967, 1981) using patterns of inactivation in X-linked genes, showed that erythrocytes and myeloid cells in female CML patients with heterozygous X-linked glucose-6-phosphate dehydrogenase (G6PDH) had the same single isoenzyme type for G6PDH in contrast to normal cells, which were heterogeneous. These results suggested that both erythrocytes and granulocytes share a common stem cell, demonstrating that CML is a clonal disease with a stem cell origin. A recent study showed that while BCR-ABL expressing progenitor cells were eliminated following imatinib treatment in patients with a major molecular response (MMR), BCR-ABL expressing HSCs were still detectable (Abe et al., 2009). In chronic phase, the leukemic clone seems to be maintained by a small number of BCR-ABL positive CD34^+^CD38^-^ cells, a population enriched for HSCs (Fialkow et al., 1977). These LSCs differentiate normally and proliferate slowly like normal HSCs. However, as these cells progress into intermediate phases of lineage restriction, their progeny proliferate losing their primitive marker CD34. By analyzing different subpopulation of primitive CML cells it has been shown that an unusual autocrine IL-3-granulocyte colony-stimulating factor mechanism can provide a strong rational for the unusual performance of BCR-ABL expressing stem and progenitor cells (Chang et al., 1989; Holyoake et al., 2002). This mechanism only moderately offsets the *in vivo* signals, which maintain normal HSCs quiescence but, when active in BCR-ABL expressing LSCs, drives their differentiation at the expense of their self- renewal. In less primitive CML progenitors, the same mechanism has a more potent mitogenic effect that is then quenched when the cells progress into the final phases of differentiation.

### Origin and Aberrations in ALL

Acute lymphoblastic leukemia (ALL) defines a group of blood malignancies that frequently have chromosomal or intra-chromosomal changes. These rearrangements can impact on immunoglobulin or T-cell receptor genes that drive commitment to the lymphoid lineage. What has been elusive is proving the existence of a rare stem cell-like population that are capable of maintaining ALL. There have been conflicting results from studies investigating whether there is a CSC-like subpopulation in ALL. The heterogeneity of ALL itself may be responsible for some of these inconsistencies. Possible explanations for the pronounced variation in response to therapy include the presence of primitive LICs for each subtype of ALL and the different biology of the cell of origin. While chromosomal abnormalities are a key hallmark of ALL, on their own they are insufficient to generate the disease. Characteristic translocations include *t*(1,19), *t*(12,21), and *t*(9,22) (Mullighan et al., 2009). Cytogenetic abnormalities and transcription profiling approaches divide ALL into several subcategories, in which prognosis and frequency differ significantly in different age groups (Mullighan, 2014). One of these subcategories is Ph chromosome-like ALL (Ph-like ALL), which gives rise to the *BCR-ABL* oncogene and this is one of the most adverse abnormalities seen in ALL patients. Ph-like ALL is the group with Ph-negative B-lineage ALL but has a transcription profile similar to those of patients that have Ph-positive ALL (Den Boer et al., 2009). Genetic abnormalities associated with ALL cases are not homogeneous, and the most common mutations associated with the Ph-like subtype are the IKAROS family zinc finger 1 (IKZF1), paired box 5 (PAX5), early B-cell factor 1 (EBF1), and transcription factor 3 (E2A) (Mullighan et al., 2007). Likewise, kinase-activating mutations are seen in 90% of the Ph-like ALL. The most frequent of these include rearrangements involving abelson murine leukemia viral oncogene homolog 1 (*ABL1*), Janus kinase 2 (*JAK2*), and FLT3 (Roberts et al., 2012).

### Origin and Aberrations in CLL

Chronic lymphocytic leukemia (CLL) is defined by a very heterogeneous clinical course. Genomic aberrations are present in more than 80% of cases including 11q deletion (11q-), trisomy 12 (11-), 17p deletion (17p-), and 13q deletion (13q-) and each of these is associated with a specific clinical outcome. Inactivation or mutation of the tumor suppressor 53 (TP53) results in a more aggressive CLL phenotype in patients with 17p- (Döhner et al., 1995). Non-mutated immunoglobulin heavy chain variable genes (*IGHV*) is linked with high-risk clinical characteristics and shorter survival (Damle et al., 1999; Hamblin et al., 1999). Using next generation sequencing techniques has revealed a more detailed panel of aberrations such as Notch homolog 1, translocation-associated (*NOTCH1*), baculoviral IAP Repeat Containing 3 (*BIRC3*) and splicing factor 3B subunit 1 (*SF3B1*) (Puente et al., 2011; Quesada et al., 2012). *NOTCH1* and *SF3B1* represent the most frequently mutated genes in CLL, being present in the majority of patients (Wang et al., 2011). The results of xenogeneic transplantation studies have shown that HSCs isolated from patients with CLL firstly differentiate into B cell progenitors and only later stimulus and rearrangements can address them within a B-CLL-like phenotype (Kikushige et al., 2011). While it’s not certain that these cells constitute a type of CSC for CLL, it does seem that further genetic/or epigenetic transformation are needed for such B cells to turn into malignant cells. Furthermore, investigations into telomere length and telomerase expression indicate that CLL cells with no mutations in the immunoglobulin heavy variable (*IGHV*) proliferate rapidly and undergo extensive cell division, which are characteristic abilities of LSCs (Damle et al., 2004). In brief, these features can lead them to leukemic transformation.

## Lscs Dependency on Hypoxia

We have previously described how LSCs can arise from HSPCs that reside in the hypoxic bone marrow niche. However, the role of hypoxia in the maintenance of LSCs is still controversial, perhaps due to point of stemness at which the hypoxia is introduced in conflicting studies, and the length and level of hypoxia (Deynoux et al., 2016; Huang et al., 2018). Nevertheless, further studies are required in order to elucidate its complex effect on LSCs maintenance and survival. Hypoxia via HIFs may drive disease maintenance and development through other mechanisms such as energy metabolism, cell cycle, quiescence, and immune function. These physiological processes can be up- or down-regulated in cancer. In the case of AML, the existence of an oxygen gradient in the bone marrow allows maintenance of primary AML cells (Griessinger et al., 2014). Rouault-Pierre et al. (2013) reported that down-regulation of HIF-2α or HIF-1α to lesser extent, induces apoptosis and prevents leukemic engraftment upon transplantation of human AML cells into mice. These results suggest that HIF-2α or HIF-1α is necessary for the maintenance of LSCs and may potentially be therapeutically targeted for AML. On the other hand, the Velasco-Hernandez et al. (2014) study reported that HIF-1α deletion does not affect mouse AML maintenance, which highlights the contradictions in the role of HIFs in AML disease. However, these differences in effect might be dependent on the particular genetic alteration that initiates the malignancy, revealing once more the enormous heterogeneity of this disease. Furthermore, Vukovic et al. (2015) developed a conditional genetic model to investigate the effect of deletion of both HIF-1α and HIF-2α during leukemogenesis. The authors showed that while HIF-2α had no influence on proliferation of AML cells in a murine model, it’s important in blocking the progression of LSCs into a malignancy. HIF-2α deletion accelerates LSCs differentiation but does not affect LSCs maintenance of AML (Vukovic et al., 2015). In CML, Zhang et al. (2012) reported that deletion of HIF-1α blocks CML progression through weakening cell cycle progression and induction of apoptosis in LSCs. BCR-ABL oncogene in CML-LICs, in fact, stabilizes HIF-1α to promote cell proliferation. Whether HIF-1α has a role in the survival mechanisms of LSCs in CLL is still unknown. In CLL, HIF-1α is stabilized even under normoxia through down regulation of von Hippel Lindau (VHL) protein, whose expression is compromised by HIF-1α microRNAs (Ghosh et al., 2009). This mechanism allows the formation of a complex (HIF-1α/p300/p-STAT3) responsible for the expression of the vascular endothelial grow factor (VEGF) (Ghosh et al., 2009). The authors indicate that up-regulation of factors such as VEGF by HIF-1α plays an important role in the microenvironment’s control of leukemic cell survival. In T-ALL, HIF-1α stabilization induces Wnt signaling through enhanced transcription of β-catenin (Giambra et al., 2015). Loss of HIF-1α decreases the LSCs frequency without affecting the growth and viability of leukemic bulk cells.

## Lscs Metabolism

Hematopoietic stem cells have a distinct energy demand to sustain maintenance. However, demand for energy and nutrients increase drastically upon cell division and differentiation. Cells in order to proliferate, on top of increasing biomass and duplicate their genome, upregulate the metabolic rates of nucleotides, proteins and lipids. Consequently, cancer cells must adapt their metabolism, particularly by increasing nutrient uptake, to maintain their uninhibited proliferation (Dang, 2012). In fact, the metabolism of cancer cells is not an indirect by-product of proliferation but also a direct reprogramming orchestrated by oncogenic signaling (Ward and Thompson, 2012; Pavlova and Thompson, 2016). Investigating the metabolic phenotype of LSCs might clarify their survival mechanism and their persistence and progression though the development of the disease. Understanding how they are metabolic distinct from HSCs could help for a better characterization of each type of leukemia.

### Glucose Metabolism in LSCs

Glycolytic flux is the main feature in the metabolism of HSCs. As a rule, HSCs are energetically dormant with active glycolysis, until they differentiate, moving to mitochondrial respiration to survive. Song et al. (2014, 2016) showed that bone marrow cells isolated from AML patients with no remission have higher expression of HIF-1α, as well as glucose transporter 1 (GLUT1) and higher expression of two of the main controlling stages of the glycolytic flux, hexokinase 2 (HK2) and lactate dehydrogenase (LDH) compared to patients with complete or partial remission and healthy donors. Bhanot et al. (2015) using metabolomics approaches demonstrated that UDP-P-Glucose, which is a glycogenic precursor of glucose, is increased in AML regardless of low levels of glycogen. Likewise, changes in glucose metabolism have been linked with clinical outcome and therapeutic resistance. Herst et al. (2011) showed that high glycolytic primary blast AML are resistant to treatment. They indicate that myeloblast glycolytic rate could be an effective and easily employed method to determine the pre-treatment prognosis of AML. This conclusion is also supported by the results of a separate study conducted in AML patients by Chen et al. (2014). Unfortunately, recent studies are not enough to profile the glycolytic phenotype of LSCs and further studies are required. In support of the above results, a study conducted in mice demonstrated that deletion of lactate dehydrogenase A (LDHA) and of pyruvate kinase and muscle 2 (PKM2), two enzymes that regulates last steps of glycolysis, reduced the chance to induce leukemia (Wang et al., 2014). Finally, CML leukocytes have higher aerobic glycolytic rates when compared to normal and CLL leukocytes (Beck and Valentine, 1953).

### Glutamine Metabolism in LSCs

An alternative source of energy is glutaminolysis, in other words the metabolism of glutamine, the most abundant amino acid in circulating blood. How tumor cells regulate the balance between glycolysis and oxidative metabolism to meet their energy need is not fully understood. While it’s been long known that the Warburg shift is a notable hallmark of proliferating cancer cells, they have an intact TCA cycle that becomes progressively more reliant on glutamine metabolism when compared to normal cells. As well as use for ATP generation, the cancer cells can use TCA cycle intermediates as precursors for biosynthetic pathways and glutamine anaplerosis can help sustain this. Furthermore, glutamine is also required for protection against antioxidants by increasing glutathione (GSH) levels that in turn neutralize ROS. In a recent study, Gallipoli et al. (2018), exploiting CRISPR-Cas9 screen, identified that glutaminase (GLS), the first enzyme in glutamine metabolism, is synthetically lethal when combined with FLT3-tyrosine kinase inhibitor (TKI) treatment. Here the authors combined complementary metabolomics with a CRISPR screen to show that glutamine metabolism, through its ability to support both the TCA cycle and GSH synthesis, becomes a metabolic dependency of FLT3-IDT AML, specifically unmasked by FLT3-TKI treatment. Knoechel and Aster (2015) recently showed that signal from the PI3K-AKT pathway shifts NOTCH-dependent T-ALL cells from glutamine metabolism to aerobic glycolysis. Using murine models and xenograft transplantation model of primary human T-ALL, the authors showed that T-ALL cells with activating mutations of NOTCH1 use glutamine as the main substrate of anaplerotic reactions that feed the TCA cycle.

### Fatty Acid Metabolism in Leukemia

As discussed in the previous paragraph, LSCs may have a high demand for glutamine to feed oxidative metabolism. Adipose tissue represents one of the major sources of glutamine in cells. The high amount of energy required from LSCs can be satisfied by using fatty acid as a fuel source. Additionally, adipocytes have a well establish role in the LSCs energy demand. Adipocytes can store energy as triglycerides, which during lipolysis can be catabolized into glycerol and free fatty acids (FFA). Thus, adipocytes may deliver FFA to cancer cells, to help meet their demands for energy and lipid synthesis. A recent study demonstrated that adipocytes provide FFA as fuel source to leukemia cells (Ye et al., 2016). Using a mouse model of blast-crisis CML, Ye et al. interestingly found that LSCs have a niche in gonadal adipose tissue (GAT). The authors used limiting-dilution transplantation assays to show that these GAT-resident LSCs gave rise to leukemia with a frequency similar to that of bone-marrow-derived LSCs. Also, the GAT-associated LSCs have high expression of the fatty acid transporter CD36. Gene expression analysis showed LSCs have a pro-inflammatory phenotype that increases lipolysis that fuels the LSCs’ high levels of FAO when compared to their more differentiated progeny or normal HSCs. These features are responsible for LSC quiescence and resistance to chemotherapy. A previous study examining primary human samples of AML identified in the CD34^+^ LSCs, a subpopulation expressing CD36, and this CD36^+^ phenotype was been linked with poor prognosis (Perea et al., 2005). In these cases, the CD36^+^ LSCs also displayed an increase in uptake of FFAs and their subsequent oxidation, suggesting that CD36 can regulate LSCs metabolism in at least a subset of human myeloid leukemia. Tucci et al. (2014) reported that ALL cells stimulate adipocyte lipolysis and use the resulting FFAs to supplement *de novo* lipogenesis and proliferation. In a separate study is shown that CLL cells, in contrast with normal B-lymphocytes, are able to catabolize lipids in order to use FFAs for oxidative respiration (Rozovski et al., 2015). FFAs can also bind the nuclear receptor peroxisome proliferator activated receptor α (PPARα). The interaction between FFAs and PPARα generates a complex that, similar to a transcription factor, activates the transcription of enzymes necessary for OXPHOS (Kersten, 2014). Adipose tissue can also be a protective compartment for LSCs during stressful condition such as drug treatment. Ehsanipour et al. (2013) report that adipocytes protect leukemia cells from L-asparaginase treatment by secreting glutamine. This is particularly relevant when considering that L-asparaginase is used in ALL treatment due to leukemic lymphoblasts being highly sensitive to the depletion of exogenous asparagine and glutamine (Oettgen et al., 1967; Kitoh et al., 1990).

### Mitochondrial Metabolism and LSCs

LSCs are resilient cells and able to exploit multiple metabolic pathways in order to survive. In fact, LSCs can, in addition to glucose, utilize fatty acids and amino acids such as glutamine in order to provide precursors of the TCA cycle to sustain mitochondrial metabolism in LSCs. Most CSCs that are dependent on OXPHOS generally upregulate this energy source. For this reason, CSCs can be sensitive to mitochondrial inhibition. IDH mutated AML-LSCs acquire increased enzymatic function generating R-2-hydroxyglutarate (R2HG) from α-ketoglutarate (α-KG), as opposed to non-mutated IDH, which catalyzes the conversion of isocitrate to α-KG. The resulting accumulation of the onco-metabolite R2HG inhibits the α-KG dependent ten-eleven translocation (TET) protein family leading to DNA demethylation which contributes to tumorigenesis (Dang et al., 2009). Furthermore, cytarabine resistant AML cells enriched in quiescent LSCs, have increased levels of mitochondrial mass, hold functional mitochondria, which is translated into increased OXPHOS levels with subsequent peak in ROS. Interestingly, even though cytarabine wasn’t effective, residual cells displayed an increased expression of OXPHOS genes together with an augmentation in FAO and upregulation of CD36 that can be predictive for treatment response in patients with AML (Farge et al., 2017). In a different study it was shown that even though AML cells have higher levels of mitochondrial mass compared to normal, they still have lower respiratory chain complex activity and lower spare respiration (Sriskanthadevan et al., 2015). Interestingly, Marlein et al. (2017) recently demonstrated that NADPH oxidase 2 (NOX2) generates superoxide that stimulates bone marrow stromal cells to transfer mitochondria to AML blast cells through AML-derived tunneling nanotubes. CLL patients also display an increased metabolic oxidative profile, which is linked to alterations in their lymphoid compartment (Jitschin et al., 2014). In this study, CLL cells were found to adapt to intrinsic oxidative stress by increasing levels of the stress-responsive heme-oxygenase 1 (HO-1). The results of this study implicate that HO-1, beyond its function as an antioxidant, has additional roles in promoting mitochondrial biogenesis and reducing the high levels of ROS present in CLL cells. A study conducted in quiescent CLL-LSCs exposed to three different stromal cell lines demonstrate that OXPHOS was significantly higher when compared to CLL cells cultured alone. Here the authors co-cultured 28 CLL patient-derived cells with bone marrow derived natural killer cells, M2-10B4 fibroblasts or HS-5 stromal cells, This study, highlights the importance of considering cell-cell interactions (Vangapandu et al., 2017). Cai et al. (2016) reported that exposure of T-ALL cells to mesenchymal stem cells (MSCs) lowered their mitochondrial ROS levels and promoted a Warburg-like shift that is characterized by an increase in glucose uptake and production of lactate with associated reduction in ATP production and mitochondrial membrane potential. In addition, T-ALL cells co-cultured with MSCs have altered mitochondrial morphology due to the extracellular signals and mediate phosphorylation of the pro-fission factor, dynamin-related protein 1 (Drp1) at residue S616. Supporting this was the observation that expression of S616-phosphorylated Drp1 recapitulates mitochondrial ROS levels, the mitochondrial dynamics, metabolic switching and chemo-resistance observed in T-ALL cells co-cultured with MSCs. A study conducted in primary lymphocytes and CD34^+^ progenitors from ALL patients indicates that since the inhibitor of mitochondrial translation, tigecycline is able to sensitize them to increased apoptosis, ALL might have higher levels of oxidative metabolism (Fu et al., 2017). Interestingly, T-ALL with higher expression of Golgi-localization of oxysterol binding protein-related protein 4L (ORP4L) are characterized by higher levels of OXPHOS compared to normal T-lymphocytes (Zhong et al., 2016). Since higher ORP4L is associated with higher OXPHOS rate and it is upregulated in 80% of CML cases, CML cells are thought to have higher levels of oxidative metabolism compared to their normal counterparts (Henriques Silva et al., 2003). This was further investigated by Kuntz et al. (2017). Performing metabolic analyses on both stem cell-enriched CD34^+^CD38^-^ and CD34^+^ and differentiated CD34^-^ cells derived from patients with CML, the authors demonstrated that most primitive LSCs have higher mitochondrial activity than more differentiated LSCs and normal CD34^+^CD38^-^ cells. Importantly, they show that primitive CML cells are reliant on higher rates of oxidative metabolism for their survival.

## Autophagy in Lscs

In the last few decades the hypothesis that LSCs, as many other CSCs, have a high-energy demand has been supported by the results of a large number of studies. High rates of metabolism also correspond to high levels of cellular stress and this can damage cellular components. In this scenario, LSCs take advantage of a survival “sustainer” process, autophagy by using it as building block provider to address their metabolic requirements. As well as this, autophagy contributes in keeping cells healthy by reducing oxidative stress. Watson et al. (2015) indicated that human and mouse HSPCs exhibited lower mitochondrial stress and increased mitochondrial clearance due to increased autophagy when compared to more differentiated cells. Interestingly, they showed that ATG genes are in chromosomal regions that in AML are frequently deleted. Mice with ATG7 and ATG5 deficiency in the HSPCs compartment developed an early leukemic phenotype that compromised animals, leading to death. Importantly, deleting autophagy in both alleles in mixed lineage leukemia-eleven nineteen (MLL–ENL) model of AML enhanced glycolysis and proliferation *in vitro* and caused a more hostile leukemia *in vivo*. Since loss of ATG genes have also been identified in other malignancies, different studies have indicated that reduction in autophagy flux represents a “plus” for tumorigenesis. However, a recent study conducted in human CD34^+^ AML cells demonstrated that low risk AML have enhanced autophagy while intermediate risk AML is associated with limited autophagy flux (Folkerts et al., 2017). To further characterize autophagy, CD34^+^ AML cells were distributed into ROS-low and ROS-high sub-fractions. AML CD34^+^ ROS-low cells exhibited higher basal autophagy and decreased survival following treatment with hydroxychloroquine (HCQ), an inhibitor of lysosomal fusion during autophagy, when compared with ROS-high cells. Furthermore, knockdown of ATG5 reduced maintenance of AML CD34^+^ cells in NSG mice. Karvela et al. (2016) provided further understanding of how autophagy inhibition affects energy metabolism of CML cells. Loss of ATG7 impaired glycolysis and induced a distinctive mitochondrial metabolism profile, and the subsequent induction of ROS encouraged differentiation of CD34^+^ CML cells in the erythroid lineage. Further investigations are still required to understand the metabolic profile of LSCs, which will also provide additional tools to elucidate the role of autophagy in sustaining leukemic metabolism.

### Mitophagy and LSCs

As described previously, mitochondrial metabolism is one of the main sources of energy for LSCs. To assure that oxidative metabolism can meet LSCs energetic demand, their mitochondria need to be conformed to meet this. We have previously indicated mitophagy as the mechanism that removes injured mitochondria and, in this context a high demand for mitophagy will probably assure the replenishment of functioning mitochondria. However, this still remains to be investigated in detail. One of the few studies conducted in leukemia regarding mitophagy is a recent study in AML (Pei et al., 2018). The authors indicate that primitive AML-LSCs have higher expression of the mitochondrial dynamics regulator F1S1 compared to non-LSCs. Using valinomycin to stress mitochondria, they show that AML-LSCs overexpressing FIS1 have higher levels of mitophagy than non-LSCs. Loss of FIS1 in AML-LSCs impairs mitophagy, leading to myeloid differentiation, block in cell cycle and restricted LSC self-renewal ability.

### Autophagy in the Initiation of Leukemia

Understanding what are the main features in the initiation of leukemia is still of high importance. Remodeling of autophagy function has been largely investigated in the transformation of HSCs to LSCs. Auberger and Puissant (2017) have recently proposed a detailed analysis of autophagy’s primary participation in leukemogenesis (Auberger and Puissant, 2017). In the case of AML, several studies have demonstrated that autophagy genes are down regulated in AML cells (Brigger et al., 2013, 2014; Walter et al., 2009). A recent study by Visconte et al. (2017), conducting sequencing of the entire exome in patients with different type of myeloproliferative disorder including AML, found that expression of most relevant autophagy genes is compromised in 14% of patients. In accordance with this, two different studies have elucidated the effect of disrupted autophagy in AML initiation and progression. Mortensen et al. (2011) demonstrated that loss of the ATG7 gene in the mice hematopoietic niche enhanced the development of a myeloproliferative disorder. Based on that, Watson et al. (2015) demonstrated that deletion of ATG5 impairs autophagy, which results in developing a myeloproliferative disease in the animals. Specifically, using a mixed lineage leukemia-eleven nineteen leukemia (MLL-ENL) AML mouse model the authors demonstrated that loss of ATG5 contributes to decreased glycolytic flux enhancing disease progression. Interestingly, they indicate that heterozygotes loss of ATG5 increased the progression of the disease while deletion in both alleles resulted premature death of mice prior to developing malignancy. Differently from AML, autophagy genes are upregulated in CML and loss of autophagy in CD34^+^ CML cells results in compromised initiation of leukemia (Rothe et al., 2014; Karvela et al., 2016). A high-energy demand is also required for leukemia expansion. Ianniciello et al. (2017) reported that primary CD34^+^ LSCs leaving hypoxic environment require metabolic adaptation to repopulate as well as for disease expansion. In the lymphoid counterpart, mRNA analysis showed that patients with CLL show increased expression of BECLIN1 and ATG5 genes than healthy controls (Kong et al., 2018). Based on these studies, autophagy seems to dictate LSC fate depending on the stage of the transformation, the type of leukemia and the presence of multiple mutations that can affect the progress in the disease.

### Autophagy in LSCs Drug’ Resistance

Several studies have shown that LSCs escape from drug treatment by upregulating autophagy. TKIs, the front-line treatment of CML, inhibit the oncogenic BCR-ABL tyrosine kinase activity and this induces autophagic flux (Bellodi et al., 2009; Helgason et al., 2013; Baquero et al., 2018). Arsenic trioxide is an alternative way to remove BCR-ABL, which requires induction of the cathepsin B, a lysosomal protease (Puissant et al., 2010; Goussetis et al., 2012). Interestingly, CML LSCs balance autophagy between a survival and apoptotic function. Autophagy is induced for BCR-ABL degradation upon TKIs treatment and at the same time promotes leukemic cell recovery following cessation of treatment (Crowley et al., 2013). Additionally, Helgason et al. (2011) highlighted autophagy inhibition with TKIs treatment as a strategic approach to eradicate LSCs. While current treatment for AML is based on combining anthracyclines with cytarabine, use of m-TOR inhibitors has been investigated. The results of these studies indicated that treatment with inhibitors of m-TOR increases protective autophagy flux in AML cells (Altman et al., 2014; Chen et al., 2017). A different approach investigated in AML is to combine conventional chemotherapy with statins, which act by restricting the last step of cholesterol synthesis (Hartwell et al., 2013). However, statins induce autophagy, which reduces the effect of treatment in targeting LSCs. A different approach tested for AML uses a recombinant arginase to deplete arginine levels in AML patients (Tanios et al., 2013). This is based on the observation that AML blast cells are dependent on income of arginine for their survival. While the effect of targeting arginine was promising in AML blasts, cytoprotective autophagy was increased. The same scenario has been indicated when the disease affects the lymphoid compartment. In T-ALL, approaches using inhibitors of NOTCH1 are used to weaken glutamine metabolism. However, NOTCH inhibitors act as a double edge sword: the effect on glutaminolysis is null since NOTCH inhibitors induce autophagy, which contributes to T-ALL metabolism (Herranz et al., 2015). Glucocorticoids are also used in the treatment for T-ALL (Jiang et al., 2015). Unfortunately, finding a cure for this disease seems to constantly run in to the same issues. Glucocorticoid effect on repressing m-TOR is translated in increased autophagy, which contributes to resistance to treatment. In the case of B-ALL, bortezomib, a proteasomal inhibitor has been indicated to increase cytotoxicity (Murata et al., 2009). However, to compensate for the loss of proteasomal activity and re-establish protein homeostasis following bortezomib treatment, autophagy is upregulated as a rescue mechanism (Milan et al., 2015; Wang et al., 2015).

## Conclusion

Autophagy is indeed an important physiological process. However, what seems the answer in certain circumstances can be the problem in others. While HSCs use autophagy to protect themselves, it can also be involved in their malignant transformation. Importantly, LSCs upregulate autophagy potentially to provide building blocks/energy in stressful conditions, such as drug treatment. The relevance of combining current treatment with autophagy inhibition in LSCs have come to of a phase II clinical trial. The name of the study is CHOICES (CHlOroquine and Imatinib Combination to Eliminate Stem cells) and combines first line treatment for CML patients with HCQ. However, HCQ is a non-specific autophagy inhibitor and high doses are required to target autophagy in patients that might not be achievable, thus there is a need to develop more specific autophagy inhibitors to target autophagy in patients. ULK1 and VPS34 inhibitors have been established and *in vitro* results are promising (Dowdle et al., 2014; Egan et al., 2015; Petherick et al., 2015; Baquero et al., 2018). However, further analysis and *in vivo* studies using suitable robust pre-clinical models are still necessary to validate their ability to target autophagy in cancer patients and specifically in the context of leukemia.

## Author Contributions

AI wrote the manuscript. All authors contributed editing and proofreading of the article.

## Conflict of Interest Statement

The authors declare that the research was conducted in the absence of any commercial or financial relationships that could be construed as a potential conflict of interest.
